# Different Hemispheric Roles in Recognition of Happy Expressions

**DOI:** 10.1371/journal.pone.0088628

**Published:** 2014-02-10

**Authors:** Akinori Nakamura, Burkhard Maess, Thomas R. Knösche, Angela D. Friederici

**Affiliations:** 1 Department of Clinical and Experimental Neuroimaging, National Center for Geriatrics and Gerontology, Obu, Japan; 2 Method and Developmental Group “MEG and EEG: Signal Analysis and Modelling”, Max Planck Institute for Human Cognitive and Brain Sciences, Leipzig, Germany; 3 Method and Developmental Group “Cortical Networks and Cognitive Functions”, Max Planck Institute for Human Cognitive and Brain Sciences, Leipzig, Germany; 4 Department of Neuropsychology, Max Planck Institute for Human Cognitive and Brain Sciences, Leipzig, Germany; University G. d'Annunzio, Italy

## Abstract

The emotional expression of the face provides an important social signal that allows humans to make inferences about other people's state of mind. However, the underlying brain mechanisms are complex and still not completely understood. Using magnetoencephalography (MEG), we analyzed the spatiotemporal structure of regional electrical brain activity in human adults during a categorization task (faces or hands) and an emotion discrimination task (happy faces or neutral faces). Brain regions that are specifically important for different aspects of processing emotional facial expressions showed interesting hemispheric dominance patterns. The dorsal brain regions showed a *right predominance* when participants paid attention to facial expressions: The right parietofrontal regions, including the somatosensory, motor/premotor, and inferior frontal cortices showed significantly increased activation in the emotion discrimination task, compared to in the categorization task, in latencies of 350 to 550 ms, while no activation was found in their left hemispheric counterparts. Furthermore, *a left predominance* of the ventral brain regions was shown for happy faces, compared to neutral faces, in latencies of 350 to 550 ms within the emotion discrimination task. Thus, the present data suggest that the right and left hemispheres play different roles in the recognition of facial expressions depending on cognitive context.

## Introduction

The capacity to recognize facial expressions is one of the most important abilities in human social interaction. It is well known that humans can easily discriminate at least six emotional expressions: happiness, surprise, fear, sadness, anger, and disgust [1], [2] (also see [3] for review). Information from facial expressions helps us to make inferences about another individual's state of mind and facilitates communication. Lines of evidence have strongly suggested that information about personal identity and emotion is, to a considerable degree, processed separately in the human brain. Likewise, different neuronal populations that are tuned to selectively respond to either identity or expression have been found in monkeys [4], [5]. Some patients with impairment of facial recognition (prosopagnosia) have shown normal performance in recognizing facial expressions [6], [7], while others have shown the opposite symptoms; impairments in recognizing facial affect but preserved ability for facial identification [8], [9]. Behavioral studies have also shown dissociation between the processing of facial identity and facial expression [10], [11]. These observations support Bruce and Young's (1986) [12] cognitive model of face recognition, which proposed distinct module-based processing pathways for facial identification, emotional expression, and speech-related facial movements. Haxby et al. (2000, 2002) [13], [14] proposed a neural model for face perception based on Bruce and Young's model, which, in principle, can explain the findings of various previous studies. The model assumes two major neural pathways, one to process invariant aspects of faces leading to facial identification, and another to process changeable aspects of faces such as eye gaze, expression, and lip movements.

Concerning the recognition of the invariant aspects of faces, various functional neuroimaging studies have shown converging results indicating that the ventral occipitotemporal region, namely the fusiform face area (FFA), plays an essential role in facial recognition [15]–[20]. In contrast, findings of studies on emotional expressions of faces appear to be more complicated. To date, several brain regions have been reported to serve important functions in recognizing facial expressions. The amygdala is one of the most important and well-known structures to be involved in the recognition of emotions, especially because of its role in fear perception, which is well established in both clinical [21], [22] and neuroimaging studies [23]–[25]. The activation of the insula in response to the disgust expression has been repeatedly reported [26], [27]. In addition, involvement of the right primary and secondary somatosensory cortices in judging emotional facial expression has been demonstrated by lesion studies [28], [29]. The inferior frontal cortex [30], [31] and the orbitofrontal cortex [32], [33] have also been reported to contribute to the processing of facial emotion. Moreover, the superior temporal sulcus (STS) region is considered important because neurons in this region are tuned to respond to social signals including facial expression, eye gaze, lip movements, and gestures [34]–[40] (also, see [41] for review). Most of these findings are supported by an extensive voxel-based meta-analysis of functional MRI studies [42]. However, because a number of distributed brain areas appear to have important roles, as reviewed above, it is still difficult to disentangle their different contributions and draw a concise and detailed picture of the brain mechanisms underlying the recognition of facial expression.

Electrophysiological studies have, to an extent, helped unravel the complex human recognition system for facial expression by providing time-series information. Various electrophysiological studies have found a prominent electromagnetic component related to face perception with peak latency around 160–200 ms (commonly known as the N170), which originates in or around the FFA [43]–[48]. Several event-related potential (ERP) studies have demonstrated that the N170 is not affected by facial expressions, but that later ERP components show expression-related changes [49]–[54]. These results suggest that the FFA, at least in a time range of around 170 ms, is not an important contributor to the recognition of facial expression, and expression-related processing takes place later than 200 ms after stimulus onset. Magnetoencephalography (MEG) can provide further useful information because of its better spatial resolution compared to electroencephalography (EEG). Using MEG, the Ioannides and Streit group demonstrated the time courses of the electrical activity elicited by emotional faces in several brain regions, including the primary visual cortex, the FFA, and the amygdala [55]–[58]. Kujara et al. (2009) [59] showed the importance of the STS in processing painful expressions. However, these sets of results are still insufficient to gain a full understanding of the brain mechanisms underlying the recognition of facial expressions. The first objective of the present study was to provide detailed spatiotemporal information related to facial expression recognition. The second objective was to analyze the possible hemispheric roles for different subprocesses of this ability. There have been two conflicting theories for hemispheric lateralization in emotional processing. The right hemisphere hypothesis posits the right predominance for recognizing and expressing emotion, irrespective of emotional valence. This theory is supported by many previous studies [6], [28]–[31], [59], [60]. The valence hypothesis states that the hemispheric roles differ depending on the types of emotion; the left hemisphere is dominant for positive emotions and the right hemisphere is dominant for negative emotions. The valence hypothesis is also supported by a considerable number of studies [61]–[64]. The detailed spatiotemporal information provided by the current study is expected to shed further light on this issue.

## Methods

### Subjects

The study was approved by the Ethics Committee of the University of Leipzig, and all subjects gave written informed consent prior to participation. Twenty right-handed volunteers (21 to 30 years old, mean age 25.3 years, 10 males) with normal or corrected-to-normal visual acuity (greater than 25/25) participated in the MEG measurements. We excluded four subjects because of noisy MEG signal or large head movements during the measurements, and analyzed data sets from the remaining 16 subjects. An additional reaction time study was conducted with ten of these 16 subjects at a later date.

### Stimuli and tasks

Visual stimuli were digitized gray-scale photos (400×400 pixels) of faces with happy and neutral expressions, both taken from 66 individuals (altogether 132 pictures), provided by the AR Face Database [65] and 66 pictures of hands with 11 different postures taken from 6 individuals [36]. The stimuli were controlled by ERTS-VIPL (BeriSoft Co., Frankfurt, Germany) and projected onto a screen using a liquid crystal video projector. The visual angle was 13°×13°, and the viewing distance was 70 cm. A small fixation point was placed at the center of the screen.

**Figure 1:**
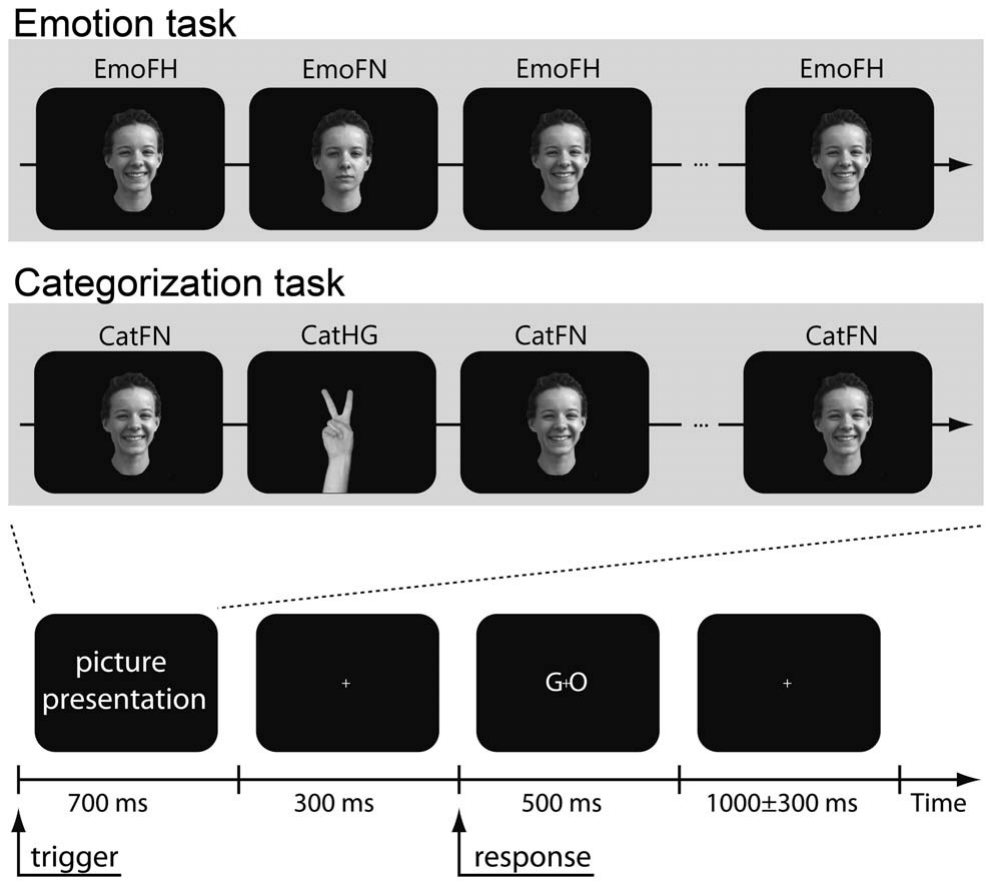
Experimental procedures. During the measurements, participants were asked to gaze at the fixation point in the center of the screen (+). First, a picture of a face either with or without expression (emotion task), or a face or picture of a hand (categorization task) was randomly presented for 700 ms. Next, a blank screen appeared for 300 ms and was followed by a “Go” signal. Then the participants had to indicate whether the face was with or without expression (or whether the picture was a face or hand) by pressing pre-assigned button 1 or 2 using the right hand. Finally, a blank screen was presented for a random time interval (1000 ms±300 ms). Then the next trial started. The assignment of the response buttons (1 or 2) was counterbalanced across participants. The photographs in the figure are not the original images used in the study, but similar images used for illustrative purposes only. The subject of the photographs has given informed consent to publication, as outlined in the PLOS consent form.

There were two different tasks. The *emotion task* involved discrimination of happy faces (EmoFH) from neutral faces (EmoFN), and the *categorization task* involved discrimination of faces (CatFH) from hands (CatHG). The CatFH picture set contained the same happy faces as EmoFH. Therefore, the contrast between EmoFH and CatFH was expected to demonstrate the differences between active and passive processing of emotional faces and reflect the influence of attention on processing facial expressions, whereas the contrast between EmoFH and EmoFN was expected to reflect the emotion-specific processing of a happy expression. Visual event-related MEG responses to both the emotion and categorization tasks were recorded in different measurement blocks, while the order of the blocks was counter-balanced across the subjects. The detailed experimental procedures are shown in Fig 1. First, a randomly selected picture was displayed for 700 ms. Selections were taken from either the set of happy or neutral faces (emotion task), or the set of happy faces or hand gestures (categorization task). Next, a blank screen appeared for 300 ms and was followed by a “Go” signal. Then, with their *right* hand, participants had to indicate via button press (pre-assigned 1 or 2) whether the facial expression was happy or neutral (emotion taks), or whether the picture was a face or hand (categorization task). Finally, a blank screen was presented for a random time interval (1000 ms±300 ms) and then the next trial started. The assignment of the response buttons (1 or 2) was counterbalanced across participants. In each task block, 396 pictures (198 per condition) were randomly presented and one measurement block took about 20 min.

Behavioral performances were recorded during the MEG recordings. Additionally, we conducted a separate reaction time study with 10 of the 16 subjects using the same task sets but without the “Go” signal. Subjects were instructed to press the appropriate response button immediately after the judgment.

### MEG recordings

A 148-channel whole-head system consisting of magnetometor sensors (WHS2500, 4D-Neuroimaging, San Diego, Ca, USA) was used for the MEG measurements. Eye movements were monitored by vertical and horizontal electrooculograms (EOGs). Signals were recorded with a bandwidth of 0.1 to 100 Hz and digitized using a sample rate of 508.6 Hz. The continuous MEG data were filtered off-line with a 0.5–30 Hz band-pass filter. In each session, 196 epochs per condition were collected and averaged in a time window of between −100 ms and +700 ms relative to stimulus onset. Direct current (DC) offset was subtracted using a pre-stimulus period (100 ms) as the baseline. Epochs with motion or eye movement artifacts (more than 30 µV in horizontal or 50 µV in vertical eye movements) or with incorrect responses were excluded from averaging.

### Data Analysis

MEG data were analyzed using the region of interest (ROI) analysis based on the principal components analysis (PCA) [36], which consisted of the following four steps: First, individual brain current source density (CSD) maps were calculated by L2-minimum-norm estimates [66]–[68] using ASA software (ANT Software BV, Enschede, Netherlands). The linear inverse was regularized using Tikhonov regularization. The regularization factor was computed according to the estimated signal-to-noise ratio [69]. For the CSD calculation, we used realistically shaped volume conductors constructed from individual MRIs. The source reconstruction surfaces were located 1 cm below the individual brain envelope, and meshed with 1222 nodes. The orientation of the current dipole at each node was free, but restricted within the surface (restricted to tangential sources).

Second, each individual MRI was spatially normalized onto Talairach and Tournoux [70] standard brain space by linear transformation. Each subject's CSD data were also spatially normalized using the identical transformation parameters. Further analyses were done within this normalized space.

Third, spatio-temporally separable independent factors in the time-series were extracted from the CSD data sets using a spatial PCA [36], [71], [72]. For the PCA, we constructed a rectangular matrix that had a column for each CSD node (1222 data points) and a row for each time instant (−100 to +660 ms, 379 points) of 3 conditions (EmoFH, EmoFN and CatFH) and 16 subjects. Since the MEG responses to hand postures had already been reported in our previous study [36] and are not a focus of the present study, we did not analyze the CatHG condition. Then the covariance matrix of the CSD was submitted to a PCA. After extraction of the spatial factors, these factors were rotated using the VARIMAX method [73]. We analyzed factors that showed an explained variance of more than 1.5% (PCA factor score) and a maximal correlation value larger than 0.5. In addition, factors that showed poor signal quality with a large amount of noise contamination were eliminated from the analyses. For this, we analyzed each PCA factor score [72] and computed the S/N ratio for (the maximal deflection in a post-stimulus time window)/(the standard deviation in the pre-stimulus baseline). Any factor that remained at an S/N ratio smaller than 10 for all conditions was eliminated from the analyses.

Fourth, an ROI analysis was performed using the spatial information from the PCA-factors in order to obtain time courses of certain brain regional electric activities. The ROIs, each of which consisted of 10 data points, were defined by thresholding as follows: To start with, we determined the data point that had the maximal magnitude in each PCA factor. Within a radius of 20 mm from that point, we then selected data points up to the 10th highest in magnitude. If a PCA factor shape involved more than two different anatomical subdivisions, we created additional ROIs starting from the local maximum within the anatomical subdivision and applied the same procedure again. For each ROI, time courses for the 3 conditions in the CSD values were calculated separately as averages across all subjects. The peak latencies were determined from the most prominent positive deflection in the averaged time courses of the EmoFH condition. If there were more than two prominent peaks, we took the earliest. The onset latency of the electric activation in each ROI was determined by scanning the magnitude of electrical activity starting from 0 ms. The latency at which the activity became stronger than five standard deviations (*SD*; uncorrected *p*<0.00001) and continued to increase for more than 20 ms was defined as the onset latency of the electric activation. For all ROIs, condition effects were tested in five time windows (TWs), which were 100 ms in length and had starting points ranging from 50 to 550 ms. The CSD values of the solutions were averaged for each ROI, condition, and TW, and were used as dependent valuables for the repeated-measure analyses of variances (ANOVAs). First, a 3-way ANOVA (ROI * condition * TW in 16 subjects) was conducted in order to check the 3-way interaction. Consequently, 2-Way ANOVAs (condition * TW in 16 subjects) were then conducted for each ROI. For the analyses, the sphericity was checked using Mauchly's test, and degrees of freedom were adjusted according to Huynh-Feldt if appropriate. Paired t-tests were applied for the post hoc analyses using the Bonferoni correction. These statistical analyses were performed using SPSS ver, 21 (IBM, New York, USA).

In order to check for the influence of individual differences in signal strength (because a few subjects with large signals may dominate the results), the PCA-based ROI analysis was performed using both normalized and original data sets. For normalization, individual CSD data sets were divided by their medians, which were calculated across all conditions, time points, and dipole positions for each subject. The outputs of both data sets were similar, but the S/N ratio was slightly better in the median-normalized data set. Therefore, the results using median-normalized data are presented in this article. Individual activation was also analyzed in each ROI. The mean activity in a 20-ms time window around the peak latency was regarded as significant when it was larger than three *SD* (uncorrected *p*<0.001) compared to the averaged baseline.

### Spatial accuracy

According to a previous simulation study [36], the spatial accuracy of source estimation of our method using the L2-minimum-norm calculation is quite reliable if a source is located close to the reconstruction surface. Most of the sources in the outer cortical mantle belong to this category. This is because MEG is most sensitive to shallow electrical sources [74]–[76]. However, due to the limitations of the 2-dimensional reconstruction, there are exceptions. These are: 1) Activity which is located in the same position but at a different depth, as, for example, the insula and frontal operculum or the FFA and cerebellum, is reconstructed into the shallow position only, i.e., the frontal operculum or the cerebellum. 2) Source activity at larger distances from the MEG sensors, i.e., in the more medial parts of the basal brain, is reconstructed with significantly less spatial accuracy (error >20 mm). Therefore, an ROI in the medial temporal region might also reflect activity from the ventral and medial temporal structure including the amygdala, hippocampus, parahippocampus, and the anterior parts of the fusiform gyri.

## Results

### Behavioral data

During the MEG recordings, all subjects were able to perform both tasks accurately. The proportions of correct responses in the emotion recognition task were 93±4% in the EmoFH condition and 94±5% in the EmoFN condition. In the categorization task, they were 97±4% in the CatFH condition and 96±4% in the CatHG condition. There were no statistically significant condition differences between EmoFH and EmoFN, or between EmoFH and CatFH. Reaction times, measured in a separate, purely behavioral study, demonstrated that the mean reaction time for the EmoFH, EmoFN, CatFH, and CatHG conditions were 558±55 ms, 564±52 ms, 410±42 ms and 413±33 ms, respectively. There were no condition differences in reaction time within each task (EmoFH vs. EmoFN or CatFH vs. CatHG). However, the mean reaction time to EmoFH was significantly longer than that to CatFH (*p*<0.0001).

### MEG data

**Figure 2:**
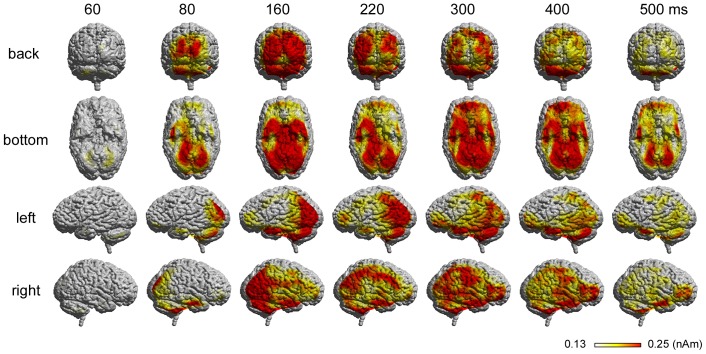
Spatially normalized CSD maps of the EmoFH condition averaged across 16 subjects. Each column shows the brain surface electric activity at a given time point. Each row shows the view from the back, bottom, left, and right.

The grand-averaged CSD maps showed spatio-temporal dynamics of brain surface electric activation while recognizing facial emotion (Fig. 2). The earliest electrical activity started at the occipital pole, and then immediately spread ventrally through the bilateral occipitotemporal regions, which were maximally activated at around 160 ms. The electric activation also showed rapid dorsal spreading. However, apparent interhemispheric differences were found, especially at latencies later than 200 ms, in that the right parietofrontal regions were more strongly activated than the left ones.

**Figure 3:**
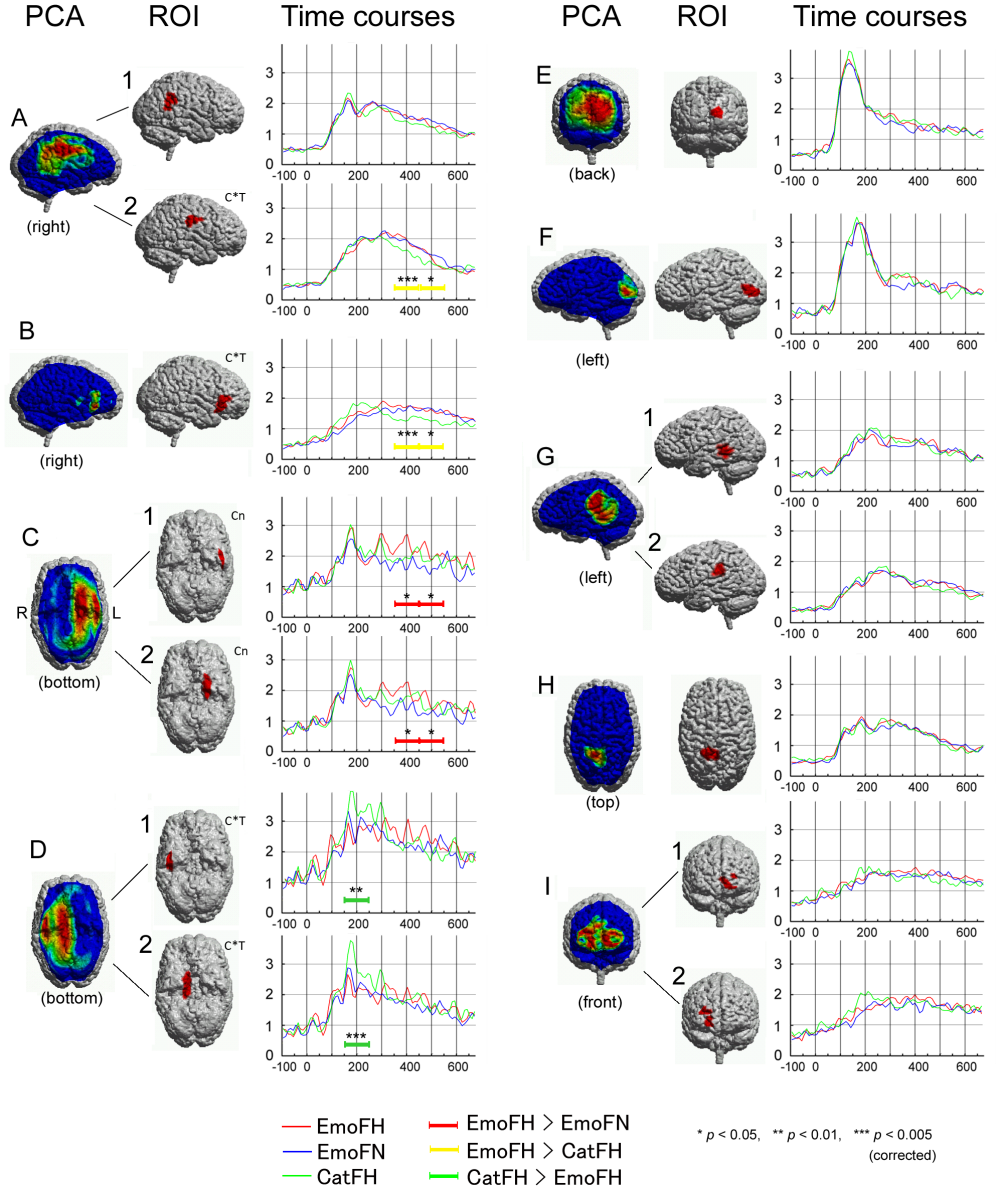
Results of the PCA-based ROI analysis. The PCA column shows the spatial distribution of nine factors extracted by PCA. Views of the brains are indicated in parentheses. The shape of each ROI created by these PCA factors is shown in the ROI column. C*T indicates ROIs that showed significant interaction (condition * TW), and Cn indicates ROIs that showed significant main effects of condition. Time courses of CSD at each ROI averaged across the 16 subjects are displayed on the right-hand side of the corresponding ROIs. Time courses of the 3 conditions are plotted in red (EmoFH), blue (EmoFN), and green (CatFH). The unit of the Y-axis demonstrates the median-normalized value. Time windows, during which statistically significant condition differences were detected by post hoc analyses, are indicated in red (EmoFH > EmoFN), yellow (EmoFH > CatFH), and green (CatFH > EmoFH) bars.

We extracted 9 PCA factors (Fig. 3, PCA, A-I) from the CSD data of the three conditions (EmoFH, EmoFN and CatFH), which accounted for 58% of the total variance. Using the spatial information of these PCA factors, we created 14 ROIs according to anatomical subdivisions (Fig. 3, ROI; Table 1). These regions were widely distributed from posterior cortices (visual) to anterior cortices (inferior prefrontal) involving both ventral and dorsal regions. The averaged time courses of all conditions at each ROI are illustrated in Fig. 3. Each of them showed a characteristic activation pattern, although the S/N ratio of the ROIs in the ventral regions (e.g., C1, C2, D1, and D2) appeared to be lower. Compared to the pre-stimulus baseline, all ROIs showed highly significant activation (at least more than 10 times *SD* larger deflections at their peak) in all three conditions. Individual data also demonstrated that at least 12 of the 16 subjects (14.4±1.6 on average) elicited significant activation in all 14 ROIs, and all 16 subjects showed significant activation in a subset of 6 ROIs (A1, A2, B, E, F, and H) (Table 1, Ind-act). The onset latency of the activation was shortest in the primary visual region (56 ms), and all ROIs showed initial significant deflections before 100 ms (Table 1, onset). On the other hand, peak latencies ranged widely from 132 ms (primary visual region) to around 400 ms (inferior prefrontal regions). The ventral occipitotemporal regions and the right posterior dorsal regions generally showed earlier peak latencies of around 160 to 190 ms (Table 1, peak).

**Table 1:** Detailed information for 14 ROIs.

PCA			Talairach	Ind-			
Factor	ROI	Brain Region	coordinates	act.	ANOVA	onset	peak
A	A1	Rt. inferior parietal	48, −45, 26	16	N.S.	74	162
	A2	Rt. central	49, −15, 30	16	*cond x TW	70	320
B	B	Rt. inferior frontal#	40, 26, −5	16	*cond x TW	78	304
C	C1	Lt. inferior temporal	−48, −9, −25	13	*cond	94	182
	C2	Lt. medial temporal#	−19, −6, −31	14	*cond	92	178
D	D1	Rt. inferior temporal	45, −10, −25	12	*cond x TW	84	164
	D2	Rt. medial temoral#	12, −10, −31	13	*cond x TW	84	166
E	E	Primary visual	16, −88, 16	16	N.S.	56	132
F	F	Lt. middle occipital	−32, −79, 5	16	N.S.	74	178
G	G1	Lt. STS	−52, −43, 1	15	N.S	82	226
	G2	Lt. inferior parietal	−52, −31, 27	15	N.S	62	310
H	H	Lt. intraparietal sulcus	−12, −48, 56	16	N.S	64	186
I	I1	Rt. inferior prefrontal	21, 50, 1	14	N.S	88	408
	I2	Lt. inferior prefrontal	−15, 54, −3	13	N.S	88	400

*Note*. Lt.  =  left; Rt.  =  right; STS  =  superior temporal sulcus; Ind-act  =  number of subjects who showed significant activation in each ROI in the 16 subjects; 2-way ANOVA column indicates the ROIs that showed significant interaction (cond x TW) or main effects of condition (cond); onset  =  latencies of activation in the time course of the EmoFH condition; peak  =  the peak latencies of activation in the time course of the EmoFH condition.

# these ROIs can detect electric activity from other brain regions (see Method, spatial accuracy).

*p<0.05, N.S.  =  not significant.

The 3-way ANOVA (14 ROIs * 3 conditions * 5 TWs) showed a significant 3-way interaction (*p*<0.05). Subsequent 2-way ANOVAs demonstrated that 4 ROIs (A2, B, D1, and D2) showed significant interactions (condition * TW), and 2 ROIs (C1 and C2) showed significant main effects of condition (Table 1 ANOVA). Post hoc analyses detected significant condition differences in 10 TWs of these 6 ROIs (Fig. 3, color bars and asterisks). Correction for the multiple comparisons were done using Bonferroni correction that p-values were multiplied by the number of condition contrasts (3) for the ROIs which showed significant interactions, and further multiplied by the number of TWs (5) for ROIs which showed significant main effects of condition. The right central and inferior frontal ROIs (A2 and B) demonstrated stronger activation in EmoFH (recognizing happy faces in the emotion task) than CatFH (categorizing faces vs. hands) in TWs 350–550 ms. On the other hand, in the left inferior temporal and medial temporal ROIs (C1 and C2), electrical activity to EmoFH was generally greater than that to EmoFN (recognizing neutral faces in the emotion task), especially in TWs 350–550 ms. In contrast, the right inferior temporal and medial temporal ROIs (D1 and D2) were activated strongest in CatFH in TW 150–250 ms.

## Discussion

Distributed brain regions for face perception and their time courses.

The present study demonstrated that a network of distributed brain regions is activated during face processing, irrespective of the task (face categorization or emotion discrimination) or condition (within these tasks: happy vs. neutral; hand vs. face). The PCA-based ROI analysis suggests that at least 9 independent factors involving 14 different anatomical subdivisions participate in face perception and facilitate recognition of the facial emotion (Fig. 3 and Table 1). This is compatible with previous studies [58], [77] and also supportive of Haxby's model [13], [14]. The regions activated are widely distributed over the brain and include the primary visual, ventral occipitotemporal, STS, parietal, frontal, and inferior prefrontal cortices, and involve most of the expected regions. Interestingly, the spreading of the electric activation over these areas appears to occur very fast. Within 50 ms after the initial activation in the primary visual cortices, all regions started to show significant activation (Table 1, onset). This is compatible with a previous study using intracranial EEG recording that reported that coherent neural activity between the FFA and other brain regions spreads very quickly [78]. On the other hand, each of our ROIs exhibited their activation peaks at quite different latencies, ranging from 130 to 400 ms (Table 1, peak, Fig. 3). This suggests that each brain region plays its own role at a particular stage of information processing. The present analysis detected activation in the left STS, which is thought to process the core changeable aspects of faces [13], [14]. However, we did not observe different activation patterns between happy and neutral faces in the STS region (Fig. 3, G1). The absence of condition effects for emotional discrimination suggests that information processing for the changeable aspects of faces, at this stage, cannot be directly associated with decoding specific emotional information. Another explanation could be response habituation. Kujala et al. (2009) [59] demonstrated that STS neurons produce reduced responses to the repetition of painful faces. The repetition of happy faces may also have led to a habituation of the STS neurons.

### Effects of attention on facial expression

Regions that showed significant task effects between the EmoFH and CatFH conditions are considered to be involved in attentive processing of the facial emotion, because the stimulus sets of EmoFH and CatFH were identical (both happy faces) but the levels of attention to facial emotions were different. Since the task effects were mainly observed in the right hemisphere, they cannot be attributed to the contamination of the motor activity related to button responses because all subjects responded using their *right* hand.

The task effects were found in the right parietofrontal network, including central (Fig. 3, A2) and inferior frontal (Fig. 3, B) regions. Since it is difficult to clearly separate the activity from pre- and postcentral cortices using MEG, the ROI A2 is considered to reflect the electrical activity from the somatosensory and motor/premotor cortices.

These findings are compatible with a number of previous studies. The right somatosensory cortex is considered to play an important role in recognizing facial expressions, because lesions affecting this area cause impaired ability to assess other people's emotional states [28], [29]. Hemodynamic studies have demonstrated the involvement of the right inferior frontal cortex in emotional processing [30], [31]. It appears plausible to explain these results using the “simulation theory”, which states that we can infer another person's intention/emotion by generating the same internal representation based on the mirror neuron network [79]–[82]. Intensive investigations have revealed that, in humans, the inferior frontal, inferior parietal, and STS regions play an essential role in the mirror neuron network [37], [83], [84]. Interestingly, the spatial distribution of the PCA Factor A (Fig. 3) appears to involve the regions associated with the mirror neuron system, suggesting that they work harmoniously during the processing of facial emotion. Leslie et al. (2004) [30] suggested that the mirror neuron system in the right hemisphere plays a key role in emotional processing. The involvement of the right mirror neuron circuit has even been demonstrated in the recognition of hand signs [36]. Therefore, it is suggested that the right mirror neuron network is important for the processing of social signals.

Recently, Monroe et al (2013) [85] reported that explicit processing of facial emotion, compared with implicit processing, elicited stronger electromagnetic activity in the left FFA in latencies of around 170 ms (M170). However, we could not detect such a condition effect in latencies earlier than 200 ms in the left ventral occipitotemporal ROIs (Fig. 3, ROI C1, C2, and F). We consider this to be due to the differences in emotion contrast. The authors found that fearful faces evoked greater M170 compared with happy or neutral faces during explicit processing of emotion. And in fact, they did not find differences between the responses to happy versus neutral faces.

### Regions involved in processing happy expressions

Previously, specific brain regions responsible for the recognition of happy expressions were not clearly identified and functional neuroimaging studies showed conflicting results. Several studies have reported activation of the amygdala in response to happy faces [23], [86]; in contrast, some other studies failed to detect any amygdala activation [25], [87].

Here, the condition contrast between EmoFH (happy) and EmoFN (neutral) was expected to help identify the brain regions and processes related to the recognition of happy expressions. Both the left inferior and the medial temporal ROIs demonstrated significantly stronger activation in EmoFH than EmoFN in the TWs from 350 to 550 ms (Fig. 3, C1 and C2). The results might reflect the amygdala activation, because it is compatible with previous MEG studies using a magnetic field tomography technique [55]–[58], [77], which consistently detected significant electromagnetic activation in the amygdala. The results are also in line with Breiter's (1996) [23] study, which demonstrated left amygdala activation by comparing hemodynamic responses to happy faces with hemodynamic responses to neutral faces. However, it is still an open question as to whether MEG is capable of detecting amygdala activity or not. MEG is most sensitive to tangentially-oriented electrical activity that is a summation of the postsynaptic potentials in the apical dendrites of thousands of pyramidal neurons arranged like palisades [74], [75], whereas such a neuronal structure is not seen in the amygdala. In addition, since the spatial accuracy of the source estimation in the medial part of the basal brain is limited, the medial temporal ROI may, alternatively, reflect activity from the medial temporal structure, including the amygdala, hippocampus, parahippocampus, and the anterior part of the fusiform gyri. (see Methods). Therefore, in this paper, we refrain from specifying the actual sources corresponding to the activity in the left medial and lateral ROIs, but do consider these ROIs to reflect the activity in the left ventral part of the brain.

### Interhemispheric differences

The present study clearly demonstrates interhemispheric differences in the brain mechanisms involved in the recognition of facial expression. Interestingly, the ventral and dorsal brain regions showed opposite patterns in hemispheric predominance for different aspects of face processing. The dorsal brain regions, especially the parietofrontal network, showed marked *right* hemispheric predominance if the subjects paid attention to the facial expression (Fig. 3 A2 and B). This is compatible with previous studies supporting the right hemisphere hypothesis [6], [28]–[31], [59], [60]. In contrast, the *left* ventral regions were strongly activated by the happy expressions during recognition of the emotional value (Fig. 3 C1 and C2), whereas the *right* counterparts were activated during categorization (Fig. 3 D1 and D2) of happy faces versus hands. In order to analyze the hemispheric differences in these left and right ventral regions, we conducted additional 3-way ANOVAs (ROI * condition * TW) for C1 vs D1 as well as C2 vs D2. For both ANOVAs, we found significant main effects of ROI (*p*<0.001 and *p*<0.05, respectively) indicating that the total CSD power in the right ROIs (D1 and D2) are generally larger than that of their left counterparts (C1 and C2).

A limitation of our study is that we did not include stimuli displaying negative emotions. Although our study does not allow us to make statements regarding negative emotions, we consider our findings supportive for the valence hypothesis [61]-[64] that the left hemisphere plays a role in the recognition of happy expressions. On the other hand, our results also demonstrated a general predominance in the right hemisphere, especially for the attentive processing of facial emotion. Thus we consider that the two theories do not pose a conflict, but are rather connectable.

## Conclusion

The present data indicate that reading of facial expressions activates a parieto-frontal network in the right hemisphere. In contrast, the recognition of the emotional value of the facial expression, for happy expressions, mainly causes activity in inferior and medial temporal regions of the left hemisphere.
